# Ethical Decision Making in Disaster and Emergency Management: A Systematic Review of the Literature

**DOI:** 10.1017/S1049023X23006325

**Published:** 2023-10

**Authors:** Joe Cuthbertson, Greg Penney

**Affiliations:** 1. Monash University Disaster Resilience Initiative, Monash University, Clayton VIC Australia; 2. Fire and Rescue New South Wales, Australia; 3. Graduate School of Policing and Security, Charles Sturt University, Australia

**Keywords:** decision making, disaster, ethics

## Abstract

Ethical decision making in disaster and emergency management requires more than good intentions; it also asks for careful consideration and an explicit, systematic approach. The decisions made by leaders and the effects they have in a disaster must carry the confidence of the community to which they serve. Such decisions are critical in settings where resources are scarce; when decisions are perceived as unjust, the consequences may erode public trust, result in moral injury to staff, and cause community division. To understand how decisions in these settings are informed by ethics, a systematic literature review was conducted to determine what ethical guidance informs decision making in disaster and emergency management. This study found evidence of ethical guidance to inform decision making in disaster management in the humanitarian system, based on humanitarian principles. Evidence of the application of an ethical framework to guide or reference decision making was varied or absent in other emergency management agencies or systems. Development and validation of ethical frameworks to support decision making in disaster management practice is recommended.

## Introduction

Decision making in disaster and emergency management guides the allocation of resources and subsequent benefits and impacts upon affected communities. Such situations present complex moral and ethical challenges at the time of event to alleviate suffering, and can also influence how allocation of funding is provided post-impact.^1^ Application of ethical decision making requires moral awareness and intent to act based on fairness and justice, the perceptions of which can vary not only based on the community affected and their respective values, but also on the individual beliefs of responding personnel. In a recently published study, a systematic literature review was completed of more than 10,000 peer-reviewed English language studies since 2000, within the context of threat assessment, sense making, and critical decision making in police, military, ambulance, and firefighting contexts.^2^ The results demonstrated that across emergency and military services personal, ethical values and moral judgment can be highly influential as to inform the potential consequence of an action, the recognition of which can potentially influence the resolve to undertake an action or not. Further, a recent research has confirmed that participating in events that conflict with personal ethics and values can result with moral injury, resulting in loss of trust and on-going feelings of severe shame, guilt, and anger.^3^ An ethical framework encompassing organizational values to support such decisions provides a basis upon which decisions and their impacts and consequences can be considered and referenced against, thereby potentially reducing the potential for conflict between organizational and personal values for decision makers facing large-scale disaster events.

The predominant focus of contemporary emergency management education usually relates to practices of managing a disaster, command and control systems, and response processes rather than ethical decision making.^4^ The application, or absence, of an ethical framework to guide decisions can influence the perceived or actual fair allocation of resources or burden of impacts. This study evaluates the explicit use of ethical frameworks or guidance applied in disaster and emergency management across different disciplines to identify ethical guidance or standards of practice applied in such settings, and if so, how.

## Aims

The aim of this study was to systematically review the use of ethical guidance to inform critical decision making in disasters and emergencies. The scope of this review is multi-disciplinary, inclusive of humanitarian care, military services, Emergency Medical Services (EMS), health care, policing, and firefighting services.

The research questions investigated included:

1. What ethical guidance exists to inform decision making in disaster management?
2. What evidence is there of application of ethical guidance in decision making in disaster management?
3. What commonality exists in ethical frameworks developed to guide decision making in disaster management?

## Methods

This systematic review was completed in accordance with Preferred Reporting Items for Systematic Reviews and Meta-Analyses (PRISMA).^5^

The research question was developed using the Patient, Intervention, Control, Outcome (PICO) standard to frame the search strategy (Table 1).^6^

**Table 1.** Patient, Intervention, Control, and Outcome

Patient	Disaster Affected Person or Community
Intervention	Decision on Response Effort
Control	No Ethical Guidance to Inform Decision
Outcome	Decision Informed by Ethical Guidance

### Literature Search Methods

#### Inclusion Criteria

The search strategy included only terms relating to or describing the intervention from Medline/PubMed (National Center for Biotechnology Information, National Institutes of Health; Bethesda, Maryland USA), CINAHL Plus (EBSCO Information Services; Ipswich, Massachusetts USA), and ResearchGate (Berlin, Germany); Table 2. All peer-reviewed statistical studies/reports detailing management of disaster and application of ethical practice, as well as consensus guidelines, protocols, or other policy statements related to management of disaster and application of ethical practice, published by government and non-government organizations, published from 2003-2022, were included. A review of the “grey literature” in Google Scholar (Google Inc.; Mountain View, California USA) was conducted using the same search terms (Table 2). This literature review was also informed by a consideration of emergency services literature, policy, and non-peer-reviewed professional journals or publications and non-medical media.

**Table 2.** Search Terms

Sources	Medline/PubMed, CINAHL Plus, ResearchGate, Google Scholar
Search Terms	Disaster *OR* Mass Casualty Incident* *OR* Emergency Management AND Ethic* AND Deci* OR Decision-making The use of * denotes all possibilities. For example, Ethic* will encompass Ethic, Ethics, Ethical, Ethically etc. Deci* will include Decide, Deciding, Decision etc.
Limits	English Language AND Published Between 2003-2022

#### Exclusion Criteria

Non-English-speaking literature, abstracts, citations, thesis, unverified or unsubstantiated press or news media reports, and articles that are not related to management of disaster and application of ethical practice were excluded.

Key data were extracted into an Excel (Microsoft Corp.; Redmond, Washington USA) spreadsheet, including: year; sample size; gender, variables assessed; study design; assessment schedule and follow-up period; analysis used; main findings and conclusions; and limitations.

#### Quality Assessment

Two review authors independently assessed all included studies for risk of bias; any disagreement was resolved by discussion. The quality of the evidence was classified into four categories according to the Grading of Recommendations Assessment, Development, and Evaluation (GRADE) approach.^7^ Of the articles assessed, the quality was varied with only two studies rated as high. Noting that the GRADE assessment provides a qualitative outcome, this approach was informative in describing perceived strengths and weaknesses of research depth that informs practice in this area.

A narrative synthesis of the literature results and reporting on key findings was completed.^6^ A narrative synthesis of findings was selected, as it has proven useful for providing a comprehensive picture of the subject matter in question.^8–10^

### Publication Currency

Acknowledging the range in publication date accepted in systematic literature reviews varies from less than 10 years, to in limited cases, more than 20 years;^11,12^ the benefit of expanding the search to a broader range of dates is limited, as research beyond this period is either superseded, referenced, or incorporated and built upon in more current studies.^11^ In accordance with this guidance, and completed in December 2022, the review included English-language papers published in the last 20 years (2003-2022) to ensure the currency of evidence. Seminal papers from outside the date range could be considered for inclusion on consensus agreement by all authors; however, none were identified in either the handsearching or review of the bibliographies and included studies.

## Results

In the identification phase of the review, the initial search strategy of databases yielded 1,772 studies for potential inclusion. Hand searching and a secondary search of bibliographies identified a further 14 studies for inclusion, providing a total of 1,786 studies.

An initial screening phase of title review was conducted by the two authors, with those either not meeting the full search criteria or outside of the defined scope excluded. A study was included for further review if initial screening could not confirm exclusion following review of the title or was not identified as a duplication. A total of 1,749 titles were excluded during this process; in total, 37 studies progressed to eligibility review.

**Figure 1.**
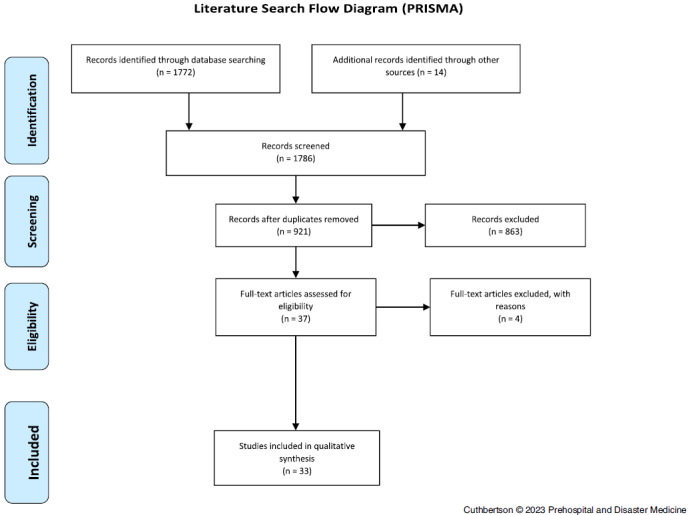
Literature Search Flow Diagram (PRISMA).

During the eligibility review, the authors initially completed a full-text review of the abstracts of the remaining 37 studies. Studies not meeting the full search criteria, or outside of the defined scope, were excluded (n = 2). Of the remaining 35 studies, one was excluded as the full English text could not be sourced, and one was excluded as it did not meet the scope of research. Any disagreement was resolved by discussion between the authors. Results are presented according to the PRISMA checklist and demonstrated on the literature search flow diagram (Figure 1 and Supplementary Material [available online only]).

Quantitative analysis was not able to be performed due to the heterogeneity of the research found in the systematic review.

The search strategy predominantly found peer-reviewed literature on health care practice in disaster, followed by humanitarian care, with limited literature found describing ethical frameworks for decision making in disaster across military, police, and fire and emergency services (Table 3). This study found evidence of ethical guidance to inform decision making in disaster management in the humanitarian system, based on humanitarian principles. Evidence of the application of an ethical framework to guide or reference decision making was varied or absent in other emergency management agencies or systems.

**Table 3.** Domains of Practice Ethical Decision Making and Disasters

Domain of Practice	Published Articles/Guidance on Ethical Practice and Decision Making in Disasters and Emergency Management
Health Care	17
Military	3
Fire and Rescue	2
Police	3
Humanitarian	5
Government	3

## Analysis

Grounded theory process was used to identify emergency themes from the collective literature. Narrative synthesis of findings was subsequently applied to explain the identified themes, as it has proven useful for providing a comprehensive picture of the subject matter in question to guide new findings and conclusions.^9,10^ The emergent themes, described in the next section of the paper, are summarized as:

1. State-based guidance is unclear.
2. Existing frameworks are siloed.
3. While Gesalt-based decision making is apparent, it is not supported by a suitable framework and may not account for future risk.

## Findings

### Theme 1: State-Based Guidance on Fair and/or Equitable Provision of Relief Funds, Resources, How These are Informed by a Human Rights or Ethics-Based Framework is Not Clear.

The development of principles to guide public health decision making in emergencies has been previously explored in the Northern American context as published by Barnett, et al in which ten principles were developed by expert consensus with a goal of linking law, ethics, and decision making for allocation of resources in emergencies.^13^ These included maintaining transparency, community participation, respecting individual rights whilst balancing community need, non-discriminatory consideration of public health needs of individuals and groups, adhering to and communicating applicable standard-of-care guidelines, identify public health priorities based on evidence, implement initiatives in a prioritized and coordinated fashion, assess the public health outcomes, ensure accountability, and share personally identifiable health information—with the patients’ consent, where possible—solely to promote the health or safety of patients or other people.^13^ Of note, these principles, whilst developed by expert consensus, have yet to be validated in practice.

### Theme 2. Identified Ethical Frameworks Exist in Siloes of Public Health, Clinical Practice, and Humanitarian Care. Ethical Frameworks for Decision Making in Military Operations are Guided by Rules of Engagement, and More Broadly, by International Law and the Conduct of Military Operations.

Literature regarding the use of triage decision making at the individual patient level is substantial and based on an ethical principle of the greatest good for the greatest number of persons. A key difference in differentiating the ethical principles of decision making related to patient care in disasters and broader emergency management decisions can be observed in relation to maintaining critical functions of society. In such circumstances, priority of decision making related to these functions may be equal to or higher than the immediate patient or health care priority it is balanced against. Kalajtzidis noted such variance in decision making theory, models, and practices, a finding that is consistent with those of Penney, et al.^2,14^ Kalajtzidis provides an overview of moral dilemma as it relates to ethical decision making in disasters, considering concepts of consequentialism and moral intuition that can inform action, but also concludes noting the unanswered question of how ethical decision should be made or guided in disasters.^14^ Ekmekci and Folayan proposed a theoretical ethical framework to guide decision making in public health emergencies, drawing on practices informed from previous studies by Beauchamp and Childress.^15^ The work of Ekmekci and Folayan was influenced by the impacts of the coronavirus disease 2019/COVID-19 pandemic to improve decision-making practices; the proposed framework would benefit from evaluation and testing in the context of an all hazards approach.^16^ Similarly, Knebel, et al proposed a decision-making process for government leadership in allocation of resources in disaster and developed a logic model utilizing a values-based framework.^17^ A focused investigation of ethical decision making of EMS in Iran undertaken by Torabi, et al revealed themes of maintaining patient dignity and respect and regulation-based actions as key tenets guiding EMS decision making.^18^ Whilst a small study and only reflective of one service, the findings of moral reasoning and decision making in the broader health care worker context is not unusual, particularly in settings of triage and resuscitation decision making.^18^

The Geneva Conventions are the international treaties that establish international humanitarian law during armed conflict. International humanitarian laws describe rules to limit suffering and provide guidance on what is and what is not acceptable during armed conflict.^19^ Thompson and Hendriks explored ethical decision making in the military context.^20^ Previous studies have found variance in military staff behavior and attitude, or have not acted in accordance with expected ethical standards.^20^ Thompson, et al focused on operational ethical conflicts that accompany decision making in military action and investigated how perceptions of harm to self and others influenced decision making through the framework of mission orders. Their findings showed moral conflict in situations where decisions would result in potential harm, but that rules of engagement provide the framework for decision making when in such situations.^20^ A key finding from this study was the recommendation of operational ethics training to support staff. The commonality between use of rules of engagement to provide a framework of decision making and EMS use of clinical practice protocols or guidelines in decision making is worth noting. In both cases, people are making complex decisions in uncontrolled environments; pre-established practice frameworks that are known and understood provide assurance to the providers to guide decision making.

In the humanitarian context of disaster response, principles of humanity, impartiality, neutrality, and independence serve as the ethical framework for decision and action. The United Nations Inter-Agency Standing Committee (Geneva, Switzerland) Operational Guidelines on the Protection of Persons in Situations of Natural Disasters were implemented to protect the rights of persons receiving disaster response or recovery interventions.^21^ These guidelines describe principles of consent, informed and involved decision making, and local ownership in respect to humanitarian response efforts.^21^ In addition to this, the Inter-Agency Standing Committee Guidelines are further strengthened by the Sphere (Geneva, Switzerland) Humanitarian Charter and Minimum Standards in Humanitarian Response.^22^ The charter core standards represent minimum criteria for appropriate care of an affected population in the humanitarian setting and is an internationally recognized set of common principles and universal minimum standards in humanitarian response.^22^ The International Committee of the Red Cross (Geneva, Switzerland) provides ethical codes of conduct for the International Red Cross and Red Crescent movements.^1,23^ Their guidance states that humanitarian aid is neither a partisan nor a political act, and must not be perceived as either; that humanitarian aid is given without consideration of race, creed, nationality, age, gender, or other qualifiers; and is prioritized based on need alone.^23^ Notwithstanding this, the provision of humanitarian care faces ethical challenges in operations as described by Clarinval and Biller-Andomo. In particular that the application of the principles are difficult, if not impossible, in some circumstances to apply in operations or that they are conflicting in settings due to lack of resources and decisions related to distribution; or, where lack of security prevents or potentially limits provision of car despite humanitarian practice operating under the international humanitarian law Rule 32: “the safety and protection from attack of objects used for humanitarian relief operations.”^24^ Clarinval and Biller-Andomo identified that whilst principles exist to guide humanitarian care, no structured reference framework currently exists that can assist aid workers in identifying potential ethical issues and support them in their decision-making process, and as such, developed and recommended a step-wise procedure to identify ethical considerations and address them in decision making.^24^

### Theme 3: Gestalt-Based Decision Making is a Feature of Health Care and Disaster Management Leadership, Yet Often Lacks an Ethical Framework to Guide it and/or is Based on A Priory Knowledge which May Not Reflect Future and Emerging Disaster Risk.

Whilst ethical guidance may exist in organizational systems, when such organizations collectively approach a singular threat, the application of an ethical framework to guide or reference decision making is clouded. In some contexts, the reference to legal authority to act on behalf of communities is evident, however, the ethical background to such decisions was based on the local judicial system and Gestalt. Krolik proposed that by incorporating a rights-based approach to disaster management, practitioners are not only ensuring that the rights of affected communities are being protected, but also that the affected communities are participating in and helping to shape the disaster management activities that impact on and involve them.^25^ Such an approach is intended to strengthen the disaster management process through involvement of community members to promote and protect human rights in disasters.^25^

Crisis standards of care are a method of decision making to inform rationalizing health care resources in a constrained environment. In such circumstances, decisions on provision and limitations of treatment are based on availability of health care resources and demand. Leider, et al systematically reviewed ethical standards that inform crisis standards of care finding that early establishment of practical guidance for invocation and application was required.^4^ Ethical frameworks for health care workers are well-established as practice norms and are commonly guided by relevant practitioner leadership bodies. Good has previously referred to this in “Ethical Decision Making in Disaster Triage” and reported the findings of Larkin and Arnold who recommended seven specific virtues to guide triage in disaster.^26,27^ Whilst the foundation of these is linked to codes of medical ethics, the validation of decision making in their application is yet to be conducted. Further to this, Berstein discussed the writing of Iserson and Moskop who had previously explored both the medical duty to respond in disaster and the ethics of application of triage when doing so. Findings showed that whilst ethical principles of duty to care and utilitarianism (greatest good) are established and known in health care, the practicalities of application and moral consequence of decision making in crisis are not well-established, and in some cases, result in legal action and prosecution.^28,29^ Similar conclusions were expressed by Holt, who noted the ethical challenges faced by medical practitioners in disasters and a need to provide education, guidelines, and a practical approach a priori to events.^30^

Erbay has critically reviewed ethical practice related to prehospital triage.^31^ Erbay reported that whilst principles of utilitarianism, beneficence, and justice underpinned triage practices, that the broad range of triage systems combined with balancing priorities of beneficence (greatest good for the greatest amount) and justice (equal chance) can result in subjective decision making.^31^ Key findings included recommendations of an ethical framework to guide application of triage practices.^31^

The predominant research identified in this review focused on analysis of practice. Lentz, et al conducted a scoping review of literature exploring moral distress in first responders. The scoping review identified a limited number of studies, and no research articles exploring moral injury.^32^ Lentz found that first responders were often faced with complex moral dilemmas and difficult ethical decisions that can result in moral distress.^32^ Studies reviewed showed that personal values guided decision making. Norberg found similar findings where first responders face complex situations requiring fast decisions with often limited or unclear information. In such situations, individuals take into consideration risks and benefits to individuals, public, and community.^33^

Papazoglou and Chopko proposed that the potential of moral harm can be decreased through preparedness, sense of control, and understanding one’s values.^34^ Likewise, Norberg recommended the importance of developing practical ethical awareness in tasks and activities;^33^ however, these factors did not decrease moral distress.^33^ Boin and Nieuwenburg focused on the consequence of an absence of guidance to inform practice, the potential and actual harm to both person requiring help and the provider.^29^ In particular, the use of discretion in overwhelming crisis situations resulting from disaster.^29^ The case example provided, the moral dilemma of the Memorial Hospital and Srebrenica tragedy, illustrates both tragic outcomes in the face of crisis and complex decision making in frontline military and health care operations.^29^ Further to this, the point is well-made by the authors of the unfair and unethical nature of resting the authority of decision making in a complex crisis and morally ambiguous situation with the first line responders attempting to manage the incident they are faced with. Consequently, the unresolved question remains of who should receive what limited resources are available, who makes that decision, and what guides it?

## Limitations

The review findings are limited due to low-quality research and lack of literature consistency. Whilst some studies may have been excluded by the search criteria, the authors took all efforts to ensure the study methodology and design were broad enough to capture all research relevant to ethical practice and guidance in disaster and emergency management, yet specific enough to ensure relevance to the contexts of decision making in this environment. A potential side effect of the highly sensitive search strategy combined with inherent limitations of the selected databases is where database searches identified studies where all or only partial search terms were present; however, the study was unrelated to ethical decision making in disaster management.

The literature retrieved via this study was high in heterogeneity, and as such, quantitative analysis was not able to be performed.

Papers not in English language was an exclusion criterion, and as such, papers not in English that may inform ethical practice in other cultures were not included.

## Implications of Key Findings

It is recommended that the establishment of a guidance framework for ethical decision making in disasters is developed and agreed upon prior to events so that actions taken are reflective of a mutually understood process. Such guidance requires the input of the communities these decisions will have consequence upon and be contextual of the respective cultural and societal values.

## Conclusion

This study found evidence of ethical guidance to inform decision making in disaster management in the humanitarian system, based on humanitarian principles. Evidence of the application of an ethical framework to guide or reference decision making was varied or absent in other emergency management agencies or systems.

## Supplementary material

For supplementary material accompanying this paper visit https://doi.org/10.1017/S1049023X23006325. click here to view supplementary material
